# Mandibular ameloblastoma treated by bone resection and imediate reconstruction

**DOI:** 10.1016/S1808-8694(15)30768-0

**Published:** 2015-10-19

**Authors:** José Raphael de Moura Campos Montoro, Marconi Gonzaga Tavares, Daniel Hardy Melo, Rosemeire de Lordo Franco, Francisco Veríssimo de Mello-Filho, Samuel Porfírio Xavier, Alexandre Elias Trivellato, André Silva Lucas

**Affiliations:** 1 Resident physician; 2 Dentist, MSc student; 3 Resident physician; 4 Dentist, MSc student; 5 Professor PhD – FMRP-USP; 6 Professor PhD – FORP-USP; 7 Dentist. Professor PhD – FORP-USP; 8 Resident physician Hospital das Clinicas da Faculdade de Medicina de Ribeirão Preto – USP

**Keywords:** ameloblastoma, segmental defects, mandible

## Abstract

Multicystic ameloblastoma mainly affects adult patients between the third and seventh decades of life, frequently in the posterior region of the mandible. The resection of a mandible segment without adequate reconstruction produces serious esthetic and functional sequelae leading to a loss of quality of life. The objective of this study is to show that multidisciplinary treatment of ameloblastomas helps in total lesion excision associated with complete reconstruction of the damaged area. We present a 47-year-old male patient with an ameloblastoma in the posterior mandible who was treated with complete resection of a mandibular segment. Reconstruction, carried out during the same surgical procedure, was performed using an iliac crest bone graft fixed with titanium plates and screws. Rehabilitation was completed eight months later with teeth implants in the grafted area. The advantages of this procedure include recurrence risk reduction due to segmental resection, reliable mandibular reconstruction and less surgical procedures, allowing full rehabilitation within a shorter period of time.

## INTRODUCTION

Ameloblastomas are rare, benign dental tumors, representing 1% of the oral tumors and cysts^1^^,^^2^. It may appear as an asymptomatic bulging or a large lesion, perforating the cortical bone, shifting tissue and causing tooth resoption1. It grows slowly and has benign appearance, local invasiveness and high recurrence rate. They may be classified as solid or multicystic, cystic and peripherical^2^. Multicystic ameloblastomas affect mainly young adults at the age of 35 years, without gender preference. It affects the mandible four times more than the maxilla, it is more frequent in the molar region and the mandibular ramus^1^^,^^3^; however it can also be found in the maxillary sinus and nasal cavity. Since it produces very little symptoms, patients usually seek care when the tumor is already large. Radiographically it shows a radiolucent uni or multilocular mass, with well outlined borders and, in most cases, associated with an impacted tooth^1^.

Treatment may vary from curettage to broad bone resections, with or without reconstruction. Radiotherapy is not indicated because the lesion is radioresistant. In the literature we also find indications for electrocauterization, cryosurgery and the use of sclerosant agents as treatment options1. Image exams are essential in the post-op follow up, because over 50% of the recurrences happen in the first five years of post-operative^1^^,^^3^.

## CLINICAL CASE PRESENTATION

**Figure 1a:**
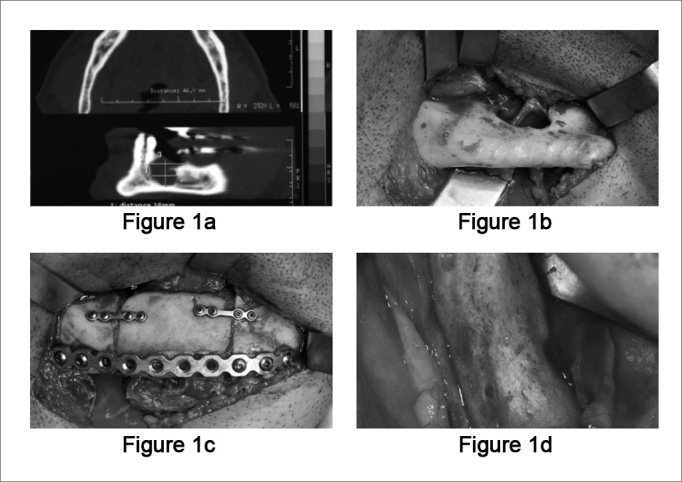
Mandible CT scan, showing the ameloblastoma destroying part of the mandibular cortical bone. **Figure 1b.** Intraoperative photograph, with submandibular access showing mandibular cortical bone erosion caused by the ameloblastoma, notice the exposed mandibular nerve. **Figure 1c.** Intraoperative photograph, showing osteosynthesis with titanium plates and screws. **Figure 1d.** Intraoperative photography (8 months after the graft), showing complete graft integration.

Patient: A.J.C., 47 years old, came to our oro and maxillofacial surgery ward at the University of Ribeirão Preto Medical School Hospital, with a bulging mass in the right side posterior mandible, without pain or inflammatory signs. His oral exam showed a local bulging, without mucosal alterations. He reported having had treatment for that tumor three years before, and the histopathology report had said it was a plexiform ameloblastoma. Radiography showed a radiolucent, unilocular lesion, of 2cm in its largest diameter, in the region of the right mandibular body. On CT scan we could better assess the tumor size and cortical bone perforation (Figure 1a). An incisional biopsy confirmed the diagnosis of a plexiform ameloblastoma. Having a definitive diagnosis and considering the possibility of a recurrence, we planned a segmentary resection with a 1cm of safety margin and immediate reconstruction with bone graft and titanium plates. Prior to the procedure, we removed the teeth involved and those near the safety margin. We fitted a passive orthodontic brace for trans-operative maxillo-mandibular block and to maintain occlusion. Under general anesthesia, we assessed the submandibular area and exposed the lesion (Figure 1b). A 2.4mm reconstruction plate was molded in the mandibular arch before resection in order to preserve facial contour. Simultaneously with the resection another surgical team removed an iliac crest bone graft. The graft was placed and fitted; it was then immobilized by the compressive action of the reconstruction plate with bi-cortical screws. In order to help keep the graft in position, we installed two more of the 1.5mm system plates with mono-cortical screws near the mandibular crest (Figure 1c). We used sutures by planes in order to close the submandibular access and that of the iliac crest. The patient was discharged on the third day of post-op, without any complications. In his first return visit we noticed facial symmetry, with good mandibular contour, intact mucosa and proper occlusion. In his post-op panoramic x-ray we noticed good graft position and size. Eight months after surgery, the patient underwent a new procedure to receive bone-integrated implants with complete graft integration (Figure 1d), thus allowing for a complete oro-facial rehabilitation, both cosmetically and functionally.

## DISCUSSION

We have presented a case of a solid ameloblastoma for which we indicated en bloc resection and immediate reconstruction with iliac crest bone graft.

Patients with ameloblastomas can be treated in many different ways. Treatment varies from enucleating and curettage to en bloc resections.^1^^,^^2^ Treatment choice depends on some factors. Multilocular ameloblastomas have higher recurrence rates than unilocular ones. Age is another important factor when considering treatment options.

The best treatment modality is still controversial. Ameloblastomas tend to infiltrate bone trabecula of the cancellous bone on the lesion's periphery, before a true bone resorption becomes radiologically evident. Therefore, the true tumor margin, often times, goes beyond the apparent clinical or radiographic margin. The attempt to remove the tumor by curettage may leave small tumor islands in the bone, which later may represent recurrences, as it is being reported in this case^1^^,^^3^.

Marginal resection is the most common treatment approach; however, we have seen reports of 15% recurrences. This technique minimizes the mandible defect; however, it can only be employed in selected cases^1^.

Many advocate a safety margin of at least 1cm beyond the tumor radiographic limits^1^^,^^3^. Others advocate segmentary resection or en bloc resection, which allows for total tumor removal and lower recurrence rates. The disadvantage of the segmentary resection is the resulting facial deformity and function loss if not properly rebuilt. In these cases, it is necessary to use grafts of flaps with bone tissue, besides implants and sophisticated surgical techniques with multidisciplinary teams. The reconstruction mode to be employed depends mainly on the defect size. Mandibular segments larger than five centimeters treated with bone grafts tend to have a higher rate of postoperative complications. Such defects must be preferably rebuilt with micro-surgical flaps from the fibula or iliac crest, among others^4^. Another alternative for large defects is osteogenic distraction.

Foster et al.^4^ reported that the vascularized bone flaps can rebuild any defect extension, while bone grafts should have their use restricted to smaller defects, less than 5cm in length. The successful use of grafts is not associated with its size only. The contact surface of its well-adjusted stumps, well-vascularized receiving bone borders, tight sealing of the oral mucosa, graft stillness with internal rigid fixation and maintenance of satisfactory dental occlusion, would all establish proper final result. Some authors believe that reconstruction simultaneous with resection brings about anatomic and functional recover, allowing the rebuilt area to be repaired in one single surgical procedure, without distortions, deviations, atrophies and scarring inherent to secondary surgeries, making this technique much more reliable^5^^,^^6^.

## FINAL REMARKS

We believe that, as long as the principles hereby described are followed, immediate reconstruction after an en bloc resection with safety margins is the best alternative to treat ameloblastomas, since it brings about total disease removal and patient cosmetic and functional rehabilitation in the same surgical procedure. Moreover, oral rehabilitation with bone-integrated implants can be done after a relatively short time, helping the patient to resume normal chewing functions.
